# 

**Published:** 1909-01-02

**Authors:** 


### BOOKS RECEIVED.

Henry Frowde and Hodder and Stoughton.

"A System of Syphilis." Vol. II. Edited by D'Arcy Power, M.B., and J. Keogh Murphy, M.D.

"A System of Diet and Dietetics." Edited by G. A. Sutherland, M.D.

P. S. King and Son.

"Philanthropy and the State." By B. Kirkman Gray.

The Surgery Publishing Co., New York.

"Practical Points in Anæsthesia." By F. Emil Neef, M.D.

Blackie and Son, Ltd.

"Our Teeth: How Built Up; how Destroyed; how Preserved." By R. Denison Pedley, F.R.C.S., L.D.S., and Frank Harrison, M.R.C.S., L.D.S.

### Resident Medical Officers' Department.

### NOTES, QUESTIONS, AND COMMENTS.

The editor of the Resident Medical Officers' Department of The Hospital invites contributions to this column both from resident medical officers and from other members of the profession. Short articles, notes, queries, or suggestions bearing upon this branch of medical practice will always receive consideration, and those that are suitable will be published in due course. Correspondence, short and to the point, is particularly invited.

Items of news from the resident officers' quarters of the principal hospitals, infirmaries, and asylums throughout the kingdom are invited, and, when of sufficient general interest, will be published. The editor will also be pleased to receive and reply to confidential communications from resident medical officers and to consider any suggestions that may be made for the improvement of this department of The Hospital.

### ANSWERS TO CORRESPONDENTS.

Rotary.—(1) The agent you mention has an excellent reputation of long standing. A personal interview is always advisable. (2) The services of an experienced accountant to investigate the books would be a good investment. (3) Your own solicitor should be quite competent to undertake the work.

